# Effective Laboratory Diagnosis of Parasitic Infections of the Gastrointestinal Tract: Where, When, How, and What Should We Look For?

**DOI:** 10.3390/diagnostics14192148

**Published:** 2024-09-27

**Authors:** Julia Dąbrowska, Maria Groblewska, Maria Bendykowska, Maksymilian Sikorski, Grażyna Gromadzka

**Affiliations:** 1 Chair and Department of General Biology and Parasitology, Medical University of Warsaw, ul. Chalubinskiego 5, 02-004 Warsaw, Poland; julia.dabrowska@wum.edu.pl; 2 Student Scientific Association, Department of General Biology and Parasitology, Medical University of Warsaw, ul. Chalubinskiego 5, 02-004 Warsaw, Poland; 3 Immunis Student Scientific Association, Cardinal Stefan Wyszynski University, ul. Dewajtis 5, 01-815 Warsaw, Poland; 4 Department of Biomedical Sciences, Faculty of Medicine, Collegium Medicum, Cardinal Stefan Wyszynski University, ul. Wóycickiego 1/3, 01-938 Warsaw, Poland

**Keywords:** gastrointestinal parasites, molecular diagnostics of parasitic infections, parasite diagnostics

## Abstract

(1) Introduction: Gastrointestinal parasites (GIPs) are one of the most common causes of disease in the world. Clinical diagnosis of most parasitic diseases is difficult because they do not produce characteristic symptoms. (2) Methods: The PubMed, Science Direct, and Wiley Online Library medical databases were reviewed using the following phrases: “parasitic infections and diagnostics”, “intestinal parasites”, “gastrointestinal parasites”, “parasitic infections and diagnostics”, and their combinations. (3) Results and Conclusions: Correct diagnosis of GIP involves determining the presence of a parasite and establishing a relationship between parasite invasion and disease symptoms. The diagnostic process should consider the possibility of the coexistence of infection with several parasites at the same time. In such a situation, diagnostics should be planned with consideration of their frequency in each population and the local epidemiological situation. The importance of the proper interpretation of laboratory test results, based on good knowledge of the biology of the parasite, should be emphasized. The presence of the parasite may not be causally related to the disease symptoms. Due to wide access to laboratories, patients often decide to perform tests themselves without clinical justification. Research is carried out using various methods which are often unreliable. This review briefly covers current laboratory methods for diagnosing the most common gastrointestinal parasitic diseases in Europe. In particular, we provide useful information on the following aspects: (i) what to look for and where to look for it (suitability of feces, blood, duodenal contents, material taken from endoscopy or biopsy, tissue samples, and locations for searching for eggs, cysts, parasites, parasite genetic material, and characteristics of immune responses indicating parasitic infections); (ii) when material should be collected for diagnosis and/or to check the effectiveness of treatment; (iii) how—that is, by what methods—laboratory diagnostics should be carried out. Here, the advantages and disadvantages of direct and indirect methods of detecting parasites will be discussed. False-positive or false-negative results are a problem facing many tests. Available tests have different sensitivities and specificities. Therefore, especially in doubtful situations, tests for the presence of the pathogen should be performed using various available methods. It is important that the methods used make it possible to distinguish an active infection from a past infection. Finally, we present laboratory “case reports”, in which we will discuss the diagnostic procedure that allows for the successful identification of parasites. Additionally, we briefly present the possibilities of using artificial intelligence to improve the effectiveness of diagnosing parasitic diseases.

## 1. Introduction

Some of the most common diseases affecting humans globally are those associated with gastrointestinal parasites (GIPs) [1,2]. However, the clinical diagnosis of most parasitic diseases is complicated by the fact they do not cause characteristic symptoms [3,4]; indeed, a correct diagnosis requires the confirmation of both the presence of the parasite and its relationship with present disease symptoms. Also, the diagnostic process must consider the possibility that infections may be caused by the presence of several coexisting parasites. As such, any diagnostic procedure should consider both the frequency of the occurrence of a parasite in a given population and the local epidemiological situation. In some cases, the presence of a parasite may not be causally associated with disease symptoms.

A key role in diagnostics is played by laboratory analysis; however, the correct interpretation of lab results requires an accurate understanding of the biology of a given parasite. Unfortunately, due to wide access to laboratories, patients often decide to perform tests themselves without clinical justification. These tests are often performed using various methods which may not be very reliable; they can frequently yield false-positive or false-negative results and are characterized by different degrees of sensitivity and specificity.

This work will review the current understanding of existing laboratory methods for diagnosing parasitic infection of the gastrointestinal tract and provide a general overview of the most frequently employed techniques. It will focus on diagnostic targets that can indicate parasitic infection, such as eggs, cysts, parasites, parasites’ genetic material, and features of the immune response. It will describe when the material should be collected, when the test can be performed to check the effectiveness of the treatment, and which material may be useful. In practice, the most frequently examined materials include feces, blood, duodenal contents, saliva, sputum, material obtained from endoscopy or biopsy, tissue sections, and other sources. It should also be noted that diagnostic findings are strongly influenced by the method of sample collection and the number of samples taken, as well as the methods used for storage, transport, preservation, and sample preparation (densification, preservation, or staining).

The paper will compare the advantages and disadvantages of the most-used parasite-detection methods. One group comprises direct methods, i.e., those used to determine the presence of the parasite in the tested material and to determine species affiliation. These may employ microscopy, and the latest molecular methods aimed at identifying parasite DNA. The other group includes indirect methods, i.e., tests whose results may indirectly indicate a parasitic invasion: such approaches can be specific, i.e., aimed at demonstrating an immune reaction in response to a parasitic infection (e.g., determining antibodies in serum), or nonspecific. All tests are subject to varying degrees of sensitivity and specificity and are prone to obtaining false-positive or -negative results; as such, all diagnostic procedures should employ various methods to ensure the most accurate diagnosis possible. 

Two other important considerations are that the chosen method should be able to distinguish an active infection from previous ones, and that it should consider the possibility of coinfection with multiple parasites. To account for the latter, the diagnostic procedure should consider the frequency of their occurrence in each population and the local epidemiological situation.

This paper will also present laboratory case reports; these will be used as the basis for discussion regarding the selection of a suitable diagnostic procedure for effective identification. It will also briefly present the possibilities of using artificial intelligence to improve the effectiveness of such approaches.

## 2. Methods

The PubMed, Science Direct, and Wiley Online Library medical databases were reviewed using the following phrases: “parasitic infections and diagnostics”, “intestinal parasites”, “gastrointestinal parasites”, “parasitic infections and diagnostics”, and combinations of these. Publications presenting the results of experimental and clinical studies, as well as review papers, were selected, which concerned the following topics: (i) what to look for and where to look for it (suitability of feces, blood, duodenal contents, material taken from endoscopy or biopsy, tissue samples, and other sources for searching for eggs, cysts, parasites, parasite genetic material, and characteristics of immune responses indicating parasitic infections); (ii) when material should be collected for diagnosis and/or to check the effectiveness of treatment; (iii) how—that is, by what methods—laboratory diagnostics should be carried out, with the advantages and disadvantages of direct and indirect methods of detecting parasites being accounted for; (iv) the possibilities of using artificial intelligence to improve the effectiveness of diagnosing parasitic diseases. Older works were cited to outline the history and introduce the reader to the presented issues. Recent papers were cited to present the current knowledge on the research problem. Additionally, laboratory “case reports” were presented in which diagnostics were performed using various methods which allow for the effective identification of parasites.

## 3. Results

### 3.1. Prevalence of Gastrointestinal Parasitic (GIP) Diseases

Gastrointestinal parasites (GIPs) can colonize the gastrointestinal tracts of humans and animals. They usually spread via the fecal–oral pathway, through ingestion of contaminated food, infected meat, water, soil, or fomites. Direct transmission is also possible, both via the person-to-person or animal-to-person routes [5,6]. 

It is estimated that about 24% of the global population is infected with GIPs. This high prevalence brings with it a greater burden in morbidity and mortality [1,6]. In most cases, the primary site of parasitic involvement is the gastrointestinal (GI) tract. The two main types of GI parasites are protozoans and helminths, with the former being more likely to be the cause of infection in developed countries than the latter. According to the World Health Organization (WHO), 1.5 billion people carry soil-transmitted helminths (geohelminths) *Ascaris lumbricoides* (roundworm), *Trichuris trichiura* (whipworm)*, Enterobius vermicularis*, *Ancylostoma duodenale*, *Necator americanus* (hookworms) or *Strongyloides stercorallis*, *Anisakis* spp., and *Trichinella* spp. [6,7]. The helminths also include the tapeworms *Echinococus* spp. and those of the genus Taenia: most cases of taeniosis have been diagnosed as *Taenia* spp. or *T. saginata*, although some examples of *T. solium* have also been noted [8,9,10,11]. 

The most common intestinal protozoal infections in developing countries are associated with *Giardia lamblia*, *Entamoeba histolytica*, and *Cryptosporidium* spp.; similarly, the most common in the United States are *Giardia lamblia*, *Cryptosporidium parvum*, *Blastocystis* spp., *Cyclospora cayetanensis*, *Cystoisospora belli*, and *Entamoeba histolytica*. Clearly, parasitic infections represent a common health issue in both developed and developing countries [12,13]. Nevertheless, over recent years, the prevalence of gastrointestinal parasitic infection in Europe has been lower than in other regions of the world. However, studies on parasitic infections are more commonly conducted in African and Asian populations, and thus less information is available regarding infections among European populations [14]. 

However, such infections are becoming more frequently encountered, with children and immunocompromised patients being particularly vulnerable. The most-diagnosed parasite in Europe is Blastocystis hominis, with a detection rate of 10.7%. Other frequently identified parasites include *Entamoeba coli*, *Endolimax nana*, and *Dientamoeba fragilis*, whose prevalence has been reported to be as high as 68.3% in some populations. Another commonly diagnosed gastrointestinal parasite is *Giardia*, with a prevalence ranging from 1.3% to 5.9%, while *Cryptosporidium* is more frequently detected in immunocompromised individuals, with a prevalence of 1.3%. *Ascaris lumbricoides* appears more frequently among individuals living in lower-income households [14,15]. 

In 2022, the 27 countries of the European Union/European Economic Area (EU/EEA) reported 731 echinococcosis cases: of these, 41% were diagnosed as *Echinococcus granulosus sensu lato*, 25% as *E. multilocularis*, and 34% as an unknown species. *Taenia solium* and *T. saginata* have much lower prevalence, estimated at approximately 0.02–0.67% [16,17,18]. Among children, the most-diagnosed parasites are *Blastocystis hominis*, *Entamoeba coli*, and *Endolimax nana*, which are primarily observed in children aged eight years and above [15].

One distinct group of patients are tourists, who are frequently identified with *Giardia lamblia*, *Cryptosporidium* spp., and *Blastocystis* spp. The Tropical and Parasitic Diseases Department in Poznan, Poland, found that 4.14% of the stool samples from 2561 tested patients, i.e., 106 individuals, were infected by GIPs, although only 50% of the tested patients reported gastrointestinal symptoms [19]. It is important to note that these infections depend on regional sanitation conditions and may vary with the season and the characteristics of the region [20].

### 3.2. Prevalence of Gastrointestinal Parasitic (GIP) Diseases—What Should Be Looked for and in What Material?

The correct diagnosis of parasitic infections remains a significant challenge due to the nonspecific nature of the clinical symptoms. Accurate diagnosis should establish a link between these symptoms and parasitic invasion. Additionally, the possibility that multiple parasitic infections may be present in a single patient complicates the diagnosis and necessitates an integrated approach.

A crucial role in identifying parasites, and thus indicating appropriate treatment, is played by laboratory diagnostic tests. However, it is essential to acknowledge the limitations of such methods, such as the possibility of false-positive or false-negative results. Again, it is also important to understand the local epidemiological situation and the prevalence of specific pathogens. 

The protozoan cysts living in the digestive system, i.e., GIPs, spread much more easily through human environments than helminth eggs: protozoan cysts are resistant to chemical water treatment agents and—unlike tapeworm or roundworm eggs—they are small enough to easily pass through water filters and enter drinking water [2]. As such, diagnostic procedures should aim to identify both invasive and dispersive forms of parasites and should include various diagnostic materials obtained from patients. 

One suitable material for study is fecal matter, as it can contain both the invasive forms of protists, primarily cysts, and their trophic forms; although the latter are generally non-invasive to the next host, they may constitute good material for identifying the parasite. In addition to feces, duodenal contents are suitable for identifying *Giardia lamblia*, and mucosa sections and blood are good materials for identifying protists, specific coproantigens, or antibodies against protists. Saliva, sputum, and urine are not used in the conventional diagnosis of GIPs but they can be a good source of genetic material for many parasites, such as *Entamoeba* spp. or *Cryptosporidium* [21].

The diagnostics for helminths are based primarily on feces examination, which may contain invasive eggs of some nematode species, as well as tapeworm segments containing many eggs or larvae, as in the case of *Strongyloides stercoralis*, *Ancylostoma*, or *Necator*. Eggs serve as both an invasive form for the next host, and a means of spreading the species throughout the environment. When searching for eggs in feces, it is important to consider the time of the analysis regarding parasite egg production, as well as fecal size and weight, as these factors will influence the choice of coproscopic method. It is also possible to detect adult worms, e.g., *Ascaris lumbricoides*, in feces. Blood tests can be performed to determine the levels of specific antibodies, as well as other parameters, such as eosinophil count. These parameters, together with coproscopy results, constitute an important basis for accurate diagnosis and the introduction of treatment. Endoscopy or biopsy materials can be taken to differentiate the causes of serious pathological processes, e.g., ulceration of the large intestine or anal prolapse caused by the presence of adult *Trichuris trichiura*, damage to the stomach wall around the cardia and pylorus, or damage to the duodenal wall induced by *Anisakis* spp. larvae ingested with fish or liver biopsies; the latter can be performed in patients with focal changes resembling hydatid cysts [22]. Cellophane swabs can be taken from the anal area as a routine test for the diagnosis of the roundworm *Enterobius vermicularis*; however, the presence of eggs or larvae in the peritoneal cavity is usually only observed in the case of a massive parasite invasion. 

Mixed invasions by different species of helminths can also occur. For example, *Ascaris lumbricoides* has been recorded with *Necator*/*Ancylostoma* or *Strongyloides stercoralis*. In addition, the patient may be infected with pathogenic protists, i.e., Giardia lamblia and *Entamoeba histolytica* or/and *Cryptosporidium* spp., or with a mixture of helminths and protists [23,24].

These processes may be further complicated by the patient’s health condition. Immunocompromised patients are more likely to demonstrate invasions by numerous parasite species from various systematic groups. The most common GIP protozoan encountered in immunocompromised hosts, especially in developed countries, is believed to be *Cryptosporidium parvum*, closely followed by *Cyclospora*; however, in less-developed countries, *Giardia lamblia* is the most common. Some opportunistic nematode species have also been noted, such as *Strongyloides stercoralis*. Unfortunately, the pathogenesis of parasitic infection remains unclear, and diagnostic testing is difficult [12,25]. 

Conventional microscopy is considered the gold standard for the diagnosis of intestinal parasitic diseases. Specimens can be obtained from feces, blood, duodenal contents, saliva, sputum, or from endoscopy or biopsy tissue samples.

**Figure 1:**
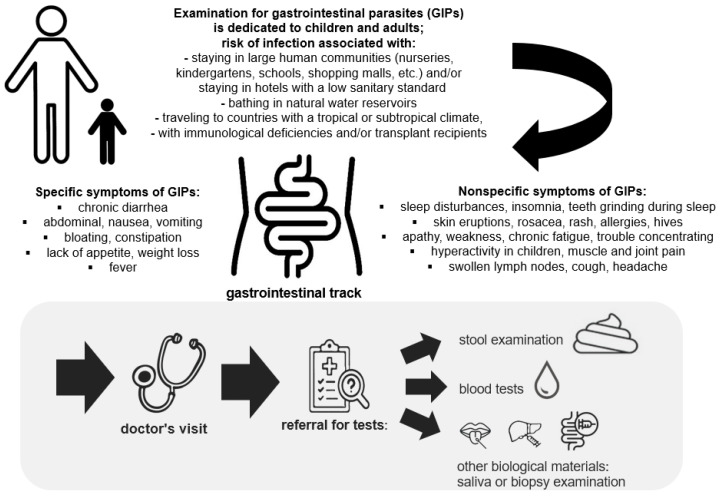
Flowchart showing the procedure from the occurrence of specific and nonspecific symptoms of gastrointestinal parasites infection, through a visit to a doctor to referral for tests using stool samples, blood, or other biological material.

A depiction of the procedure from the occurrence of specific and nonspecific symptoms of gastrointestinal parasites infection to a visit to a doctor to referral for tests using stool samples, blood, or other biological material is presented in Figure 1.

#### 3.2.1. Feces

A diagnosis of gastrointestinal parasitic (GIP) invasion is typically achieved by microscopic examination of stool samples [26]. Such coproscopy is used for the detection of protozoan cysts and trophozoites, as well as the eggs, larvae, and adult forms of helminths [27,28,29], and offers relatively low cost and wide accessibility. Though considered the gold standard for the diagnosis of intestinal parasites, coproscopy is time consuming, and highly dependent on the skills and experience of the microscopist [30]. The method also fails if the number the parasites is very low, or if the stadium cannot be demonstrated due to the life cycle in the host. Also, the technique has lower sensitivity and specificity than molecular methods, and cannot differentiate between some pathogenic and non-pathogenic species, like *Entamoeba histolytica* and *E. dispar* [31,32]. Therefore, microscopy is typically implemented in combination with other methods to obtain more reliable results [33,34,35]. 

The presented review of coproscopy methods was developed based on over 50 publications that have been published in the last 20 years. It includes standards and recommendations regarding the performance of coproscopic tests in patients suspected of GIP infection; these are all available on the websites of diagnostic laboratories and in scientific journals.

Feces should be gathered in a special container labeled with the patient’s name and surname, as well as the date and time of collection. Samples should be obtained before the start of any medication, or one–three weeks after discontinuation of the medication in the case of antibiotics or antiparasitic drugs. The specimen must not be contaminated with soil, water, or urine, and should take up to 2/3 of the container’s capacity. It is advised to collect samples from three different parts of the feces to maximize the chances of parasite detection. As the biological rhythm of the parasite is influenced by certain environmental factors like temperature, humidity, time of day, or season [36], the stool samples should be collected over a period of 10 days, at two–three-day intervals, to increase the likelihood of detecting parasites, especially protozoan cysts which are excreted irregularly. However, it is difficult to find evidence that would confirm the significance of certain factors, like the phase of the moon, on parasite reproduction. Samples should be transported to the laboratory as quickly as possible; they should be maintained at room temperature or cooled in the case of coproantigen detection. 

The macroscopic examination should evaluate the consistency of the stool (formed, soft, loose, watery), as well as the presence of mucus, blood, or fragments of parasites (e.g., *Taenia* spp. proglottids). The microscopic evaluation is usually performed using the 10× objective and should be performed systematically, starting from the corner of the coverslip, working along to the opposite corner in a straight line, and then moving one row aside. When parasite structures are found, the objective should be changed to 40× to examine in more detail [37].

The most basic and easiest way to perform microscopic testing involves the use of wet mounts, which are employed in the initial phase of the diagnosis. The World Health Organization (WHO) recommends they are performed in every laboratory. The samples can be unstained, as in the case of a saline wet mount, or temporally stained with iodine, for example. As noted above, the 10× objective is typically used, with the 40× objective being used to observe any suspicious objects in greater detail [38].

Alternatively, a direct wet mount can be used. According to Khanna et al. (2014), this procedure has five key advantages: (i) it is a fast, simple procedure and provides a quick answer when positive; (ii) it provides an estimate of the parasitic burden; (iii) it can be used with unpreserved specimens to detect the characteristic motility of the trophozoites; (iv) it can be used as a safeguard, as some protozoa may at times not concentrate properly because of unknown factors; (v) it can detect the motile trophozoite stage of the protozoan species [38,39,40]. 

A saline wet mount is made by mixing a small volume of stool with a drop of physiological saline. It is a routine, affordable test often performed in a hospital setting to achieve prompt diagnosis. It can be used to demonstrate the motility of the trophozoites, but it is rarely sufficient to establish a definite diagnosis, since the internal structures are poorly visible [39]. An iodine wet mount is prepared by placing a small drop of saline and mixing it with a small drop of iodine solution followed by adding a volume of stool the size of a match head. The iodine wet mount method aids in differentiating and identifying parasites by highlighting characteristic morphological features and internal structures. Iodine also stains the nuclei and the glycogen, which enables the detection of protozoan cysts. One of the disadvantages of this technique is that the preparation dries within a few minutes. This can be solved by combining it with glycerol [39,40]. Alternatively, if the presence of protozoan trophozoites is suspected, a buffered methylene blue wet mount can be prepared by placing a large drop of buffered methylene blue and mixing it with a small volume of feces; this approach stains the internal structures of trophozoites and cysts [38,39]. 

If an infestation cannot be detected by direct wet mount techniques due to a low number of parasites in the specimen, the sample can be subjected to routine concentration techniques, such as sedimentation and flotation. Both are used to concentrate helminth eggs and larvae, protozoan cysts, and *Cyclospora*/*Cystoisospora*/*Cryptosporidium* oocysts; however, protozoan trophozoites cannot be recovered by these methods, which makes the direct wet mount method obligatory. 

In sedimentation, a specimen is diluted with a solution of a lower specific gravity, resulting in any parasitic organisms concentrating as a sediment at the bottom of the flask. In flotation, on the other hand, solutions of higher specific gravity than the parasites are added, resulting in their flotation at the top of the solution [41,42]. One sedimentation approach that can be used to recover all parasites present by centrifugation into a fecal pellet is the formalin-ethyl acetate (FEA) method. Briefly, a small amount of specimen is strained through two layers of gauze into a centrifuge tube, and 0.85% saline or 10% formalin is added. The sample is then mixed and centrifuged and debris and fat are extracted with ethyl acetate. After the procedure the parasites are left in the sediment at the bottom of the tube. The sediment is then examined as a wet preparation, using 10× and 40× objectives, with or without iodine [32]. Sedimentation techniques are recommended for general laboratories, since they are easier to perform and carry a lower risk of error. 

Flotation techniques are suitable for separating most parasites from fecal debris. The most widely used flotation solutions are zinc sulfate solution and sodium chloride. This technique results in a cleaner wet-mount preparation than sedimentation; however, some heavy eggs like infertile Ascaris eggs, *Fasciola*, *Clonorchis* spp., or *Opisthorchis* spp. will sink and be lost with the sediment. Nevertheless, protozoan cysts or Schistosoma mansoni eggs have been successfully detected by this method [43]. It is important that flotation should not be prolonged for longer than recommended, because “light” eggs and cysts will eventually absorb water, fall to the bottom, and mix with the debris [42,44].

The McMaster flotation technique can be used to count the number of parasitic elements per gram of feces, viz. eggs per gram (epg), oocysts per gram (opg), cysts per gram (cpg), and larvae per gram (lpg), using a McMaster slide. This special counting chamber is filled with 2 g of fecal suspension and diluted in a measured amount of flotation solution, thus allowing the calculation of the number of eggs per gram of feces [44,45]. The McMaster method is extensively used to assess the levels of soil-transmitted helminths, like *Trichuris trichiura*, *Ascaris lumbricoides*, and hookworms. It is often used in combination with the Kato–Katz and the mini-FLOTAC method and has been compared with them. Levecke et al. report it to be a robust method, with an accurate multiplication factor, and to demonstrate reliable efficacy and accuracy; it can also be easily standardized [46,47].

The Mini-FLOTAC is a logical evolution of the FLOTAC, which was designed to perform a very accurate multivalent, qualitative, and quantitative diagnosis of parasitic eggs, larvae, oocysts, and cysts in fecal samples [44]. It can be applied in the diagnosis of protozoan and helminth infections in humans and animals. However, the procedure requires centrifugation and might therefore be out of the reach of resource-constrained settings [48]. Briefly, the stool is combined with the flotation solution in a special cylindrical device with two flotation chambers, which allow up to 1 g/2 g of stool to be prepared for microscopic analysis. This method demonstrates higher sensitivity and accuracy than the McMaster and the Kato–Katz methods, despite the latter two being more popular. All FLOTAC techniques can be performed on fresh fecal material, as well as preserved stool samples, and require approximately 12–15 min of preparation time before microscopic analysis [48]. It is suitable for detecting *Strongyloides stercoralis* and intestinal protozoa like *Giardia lamblia* or *Entamoeba histolytica*/*dispar*/*moshkovskii* [49]. 

The Kato–Katz method is the most-used technique for the detection of soil-transmitted helminths. Briefly, a 40–60 mg stool sample is placed on a microscope slide and covered with a hydrophilic cellophane previously soaked for 24 h in a glycerol–malachite green solution. The sample is pressed to spread the feces evenly and left for 20–30 min; then, the sample is examined under the microscope. The Kato–Katz method has been found to demonstrate high specificity for the diagnosis of *Ascaris lumbricoides* or *Trichuris trichiura* infections: previous studies only detected low numbers of false-positive readings: 0.35% for *T. trichiura* (*n* = 5) and 0.28% for *A. lumbricoides* (*n* = 4) [50,51]. The test was also found to demonstrate 91.7% sensitivity for the detection of *S. mansoni* based on four smears. Although the Kato–Katz concentration method generally has a higher sensitivity than direct microscopy and is semi-quantitative for *A. lumbricoides* and *T. trichiura*, it is more time-consuming and relies on collection of multiple stool samples to maximize sensitivity [52,53]. However, increasing evidence suggests that the sensitivity of the Kato–Katz method may in fact be lower than expected, and other, more modern techniques are under evaluation [54]. Permanent stains are not routine tests; however, they can support a more definite diagnosis by enhancing the characteristics of certain organisms. The stains are examined under oil immersion (100×), and a minimum of 300 fields should be examined before the result is determined to be negative. Permanent stains are not recommended for the identification of helminth eggs and larvae. These organisms often stain too darkly or are distorted, making identification difficult [32]. 

The most popular staining methods are the trichrome, iron–hematoxylin, and Ziehl–Neelsen techniques. Trichrome stain can be used for the detection of amoebae, including *Entamoeba coli*, *E. histolytica*, *E. hartmanni*, *E. nana*, and *Giardia lamblia* [55]. Iron–hematoxylin has been used to study the morphological characteristics of Blastocystis hominis and has shown great success in the detection of *Dientamoeba fragilis* [56,57]. Ziehl–Neelsen staining is employed in the diagnosis of *Cryptosporidium* spp. but was recently proven to be a very good method for the differentiation of *Taenia saginata* and *T. solium* eggs [58,59].

Over the last decade, many DNA-based parasite-detection methods have been published. However, fecal samples are among the most complex specimens for direct PCR testing because of the presence of PCR inhibitors such as heme, bilirubin, bile salts, and complex carbohydrates, which are often coextracted along with pathogen DNA [60]. Although recent approaches can eliminate many fecal inhibitors, they include multiple expensive, time-consuming steps, and can only process a limited number of samples at the same time. Also, the transport of fecal samples at ambient temperature can lead to rapid degeneration of parasite DNA, especially for highly labile stages such as trophozoites. To counter this, DNA samples may be preserved by refrigeration or in polyvinyl alcohol (PVA), fixative sodium acetate–acetic acid–formalin (SAF) or formalin [61]. However, these methods may decrease the sensitivity of PCR over time. Therefore, it is recommended to freeze fresh fecal specimens at −20 °C before extraction of DNA to avoid reducing the sensitivity of the molecular methods.

**Figure 2:**
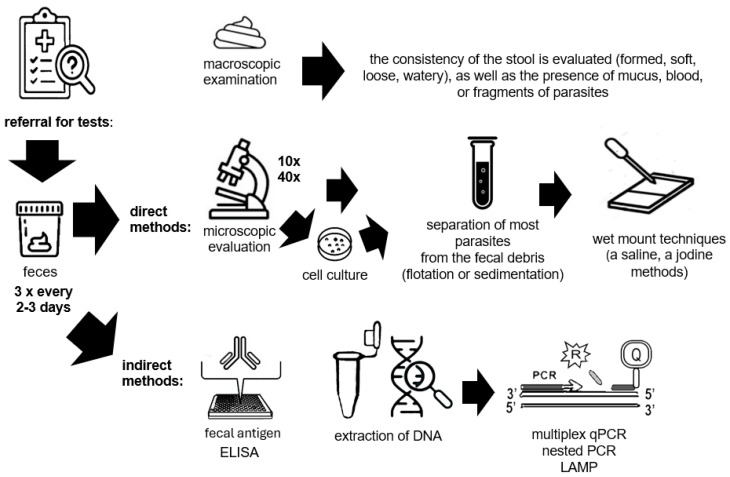
A diagram showing the procedure from referral for diagnostic tests through stool colletion to performing various diagnostic tests using direct and indirect methods. The description of the methods can be found in the text above.

The flowchart showing the procedure from referral for diagnostic tests through stool collection to performance of various diagnostic tests using direct and indirect methods is presented in Figure 2.

#### 3.2.2. Blood

Blood is frequently used for the diagnosis of parasites. While it is often directly tested for the presence of blood protozoa, for example in the case of malaria, babesiosis, trypanosomiasis, or filariasis, it is rarely used in case of intestinal parasites. A thin blood smear can be used to determine parasite morphology, but this approach is not suitable where parasite density is low; in such cases, a higher probability of parasite detection is afforded by a thick blood smear, which includes much more concentrated blood elements [62]. Although the thick blood film is more sensitive, and saves time during the examination, the thin film technique carries less risk of parasite distortion and permits parasite identification when the thick film cannot be performed [38]. Though helpful in the diagnosis of blood diseases, blood smears are rarely useful in the detection of gastrointestinal parasites (GIPs).

One of the most common initial steps in the diagnosis of parasitic infection is the identification of peripheral eosinophilia, which can be caused by parasitic infection of nearly any bodily tissue [63]. However, while eosinophilia can be confirmed by blood samples, they should be collected with special caution, since several viral, bacterial, and parasitic infections can be transmitted with the blood. Blood testing should always occur before the start of treatment. In cases of diseases like malaria, the specimen should be collected and examined immediately, but if leishmaniasis or filariasis is suspected, it is not necessary to collect the material as quickly. Also, the amount of material depends on the type of suspected parasitic invasion. The sample should be drawn from capillary blood, and both thin and thick smears must be performed, each two or three times. The smears ought to be prepared up to 30 min after collection. The specimen is examined as a direct smear in physiological saline to observe moving trypanosomes and microfilariae; other tests include thin blood smear, thick blood smear, parasitemia assessment, Knott’s concentration, or staining using Giemsa stain or H&E (hematoxylin and eosin) stain. Microscopic examination of the thin smear should be performed under the 100× objective and the thick smear under the 100× objective or higher [37].

When the species density is too low for the use of traditional methods, parasites can be identified indirectly using serology tests employing blood specimens and feces samples, in this case, called coproantigens. When combined with microscopic observation, this approach can provide faster and more insightful diagnosis. Some commercially available immunoassays are combinatorial, i.e., they simultaneously test for more than one species; for example, one test detects *Giardia* spp. and *Cryptosporidium* spp., which frequently cause coinfections in hosts [26]. The most widely used serology tests include the enzyme-linked immunosorbent assay (ELISA), the hemagglutination (HA) test, indirect or direct immunofluorescent antibody (IFA or DFA) tests, the complement fixation (CF) test, and immunoblotting. 

The main advantages of serology include its simplicity, relatively low cost, and short waiting time. Serology can also distinguish recent from past infection by assessing the titer of both IgG and IgM antibodies and antibody avidity [64]. Also, serology allows infection to be diagnosed in an early stage, when microscopy can still be negative: serological testing for antibodies against Fasciola antigens has high sensitivity and can be used during the acute phase, long before eggs appear in stool [65]. Also, such tests can be later repeated after administration of the medication to monitor treatment effectiveness, and the results are easier to interpret. However, one disadvantage of serology is that the diagnosis is retrospective, as the levels of antibodies vary in the period after infection [26,66]. While the antigen may be present in the blood before seroconversion, it may not be accompanied by antibodies, which can make the diagnosis impossible. 

ELISA (enzyme-linked immunosorbent assay) is the most popular serology technique used for detection and quantification of viral, bacterial, and parasitic antigens. It is characterized by greater sensitivity and specificity than microscopy. It is often performed to diagnose species like *Fasciola hepatica*, *Cryptosporidium parvum*, *Echinococcus granulosus*, *Trichinella spiralis*, and *Strongyloides stercoralis* [63,67,68,69,70]; it was found to be an effective method for detecting Cryptosporidium, with sensitivity, specificity, and positive and negative predictive values of 98.86%, 94.32%, 89.69%, and 99.40%, respectively [67]. ELISA was also effective in the diagnosis of *Fasciola hepatica*, where it demonstrated 100% specificity and sensitivity [71]. 

ELISA is also the most popular technique for the diagnosis of trichinellosis. The clinical symptoms of this disease are nonspecific, consisting mainly of fever, facial edema, and myalgia, and are accompanied by biological signs, such as high eosinophil count and increased muscle enzyme levels (e.g., creatine phosphokinase and aldolase). As these symptoms mimic those of influenza, dermatomyositis and other parasitoses, such as toxocariasis and schistosomatosis, serologic tests play an important role in the diagnosis [72]. Human trichinellosis can be diagnosed by a combination of medical history, clinical presentation, laboratory findings, and through the detection of anti-Trichinella IgG in host serum. 

The most common approach for detecting *Trichinella* spp. antibodies currently is ELISA together with indirect immunofluorescence [73]. However, although the method has high sensitivity, it also carries a high risk of cross-reaction. In response, Western blotting is often employed to confirm the diagnosis, with a bead assay that can detect and quantify total IgG or IgG4 *Trichinella* spp. antibodies in human serum using T. spiralis excretory–secretory (E/S) antigens [74]. *Trichinella spiralis* excretory–secretory (ES) antigens are proteins, glycans, lipids, and nucleic acids that affect host tissue and immune cells and allow the establishment of the parasite in the host by facilitating penetration, migration, nutrition, and survival [75,76]. The method had good performance, especially in cases when multiplexing is involved. An Iranian research group found ELISA to have better sensitivity (93.5%) than IFA (87%). Although both techniques showed false-positive results, these were less common with the ELISA assay. However, one of the major drawbacks of these tests is their considerable cross-reactivity with other nematode species, particularly with filarial infections [75]. 

Another serodiagnostic technique is hemagglutination. This test is used to detect the presence of specific antibodies based on agglutination caused by the antibody binding to the erythrocytes. Although the indirect hemagglutination test is primarily used to diagnose viral infections and blood types, it was found to be effective in diagnosing cystic echinococcosis, with a sensitivity of 88% and specificity of 98.4% [76]. Also, IHA and ELISA have been found to be effective diagnostic tools for cystic echinococcosis [77]. Indirect or direct immunofluorescent antibody assays (IFAs or DFAs) and counter immunoelectrophoresis and indirect hemagglutination were each 100% effective in all cases of invasive amebiasis [78]. 

IFAs and DFAs use fluorescent microscopy to detect antibodies to specific antigens. The direct assay uses only one type of antibody: this forms a complex with a fluorophore, which then binds to the antigen. In contrast, both forms of IFA use two sets of antibodies: a primary antibody that binds the antigen and a secondary antibody that binds to both the primary antibody and the fluorophore. This technique is more sensitive than direct immunofluorescence, because multiple secondary antibodies can bind to the same primary antibody. The increased number of fluorophore molecules binding per antigen increases the amount of emitted light, and thus amplifies the signal. In IFA, only one fluorophore can be attached to the antibody which lowers the specificity but reduces the chance of cross-reactivity. IFA has shown higher specificity than parasitological tests in the diagnosis of Strongyloidiasis [79,80]. Studies have shown IFA to be a useful, but expensive, method that can be performed to confirm a diagnosis established by microscopy. 

Another serodiagnostic test used in the diagnosis of parasitic diseases is complement fixation (CF), which can detect certain antigen or antibodies in serum. However, it is often replaced by the more popular ELISA and IFA tests. Wilson et al. (1977) found IFA to have 95% sensitivity and 69% specificity and CF 98% and 100%, indicating that IFA is the superior test, with much higher sensitivity but similar specificity [81].

Immunoblotting, also known as Western blot, is a highly sensitive serological technique. Unlike other serological techniques, it can be used to detect different fragments of an antigen [82]. Abuseir et al. (2013) report that immunoblotting can effectively detect *Taenia saginata* in infected cattle; this can significantly improve the process of testing meat for *T. saginata* infection [83].

Other studies have also evaluated the use of immunoblotting in the diagnosis of trichinellosis [84,85]. Although ELISA and IFA are commonly used in the diagnostic process, they have a high risk of cross-reaction with antigens of other parasites. Therefore, Western blot is often used to differentiate the findings [86]. One study described the development and specificity of commercial Western blot strips made with crude Trichinella antigens [86,87,88]. Non-commercial Western blots were found to be largely inferior to commercial blots, as the latter yielded a larger number of sharper bands [72].

#### 3.2.3. Other Biological Material Used in the Diagnosis of Parasitic Diseases

A diagnosis of gastrointestinal parasites can be also achieved using other biological materials. Saliva, urine, and sputum are used as sources of DNA for molecular diagnostic tests. They can be obtained more quickly than blood biomarkers, can be obtained non-invasively, and are hence more frequently used for a quick diagnosis or treatment monitoring [89]. Saliva is listed as one of the acceptable materials for molecular diagnostic tests. Research suggests that over 70% of salivary DNA comes from leukocytes present in the specimen [90]. Samples should be obtained at a time recommended by a doctor. If instructions are not given, patients ought to collect the saliva in the morning, before washing their teeth, or at least 30 min after ingestion of any food or fluid. Sterile swab is placed in the oral cavity, chewed, or kept beneath the tongue, until the patient cannot restrain swallowing any longer. Next, the swab is placed in a test tube and closed with a lid. Other methods include the use of Salivette swab—a saliva collection device. It contains a sterile cotton swab that is placed in the oral cavity and chewed for 45 s. Chewing stimulates production of saliva that is absorbed by the swab. The swab is then placed in a special container and centrifuged. The process leaves a clear sample, without the debris or mucus that are captured during the centrifugation process [91]. Needham et al. report that saliva showed potential as a biomarker of infection intensity in community diagnosis of the nematodes *T. trichiura* and *A. lumbricoides*, as well as hookworms, using specific IgG antibodies [92]. Imaging techniques such as ultrasound (US), computerized tomography (CT), or magnetic resonance (MRI) are often performed as an initial step in the diagnosis and staging of cystic echinococcosis. Ultrasound plays a fundamental role in parasite detection, but MRI was proved to be superior to CT in cases when US cannot be performed [93]. These techniques are also helpful in the diagnosis of cysticercosis, where CT is considered the optimal approach for visualization of cysticerci in soft tissues, appearing as small calcifications, but MRI demonstrates greater sensitivity when identifying cysts in neurocysticercosis [94,95]. Ultrasound and magnetic resonance cholangiopancreatography (MRCP) can be used to observe juvenile and mature F. hepatica within the gallbladder and common bile duct [96]. Biopsy can also be appropriate, particularly when non-invasive diagnostic methods are inconclusive; it may be very useful for the definitive diagnosis of cystic echinococcosis, cysticercosis, and even in cases of giardiasis when the trophozoites attached to the intestinal wall can be detected [97,98,99]. Biopsy samples (minimum 2 × 2 mm) should be placed in a sterile container and secured from drying out by placing them in 0.9% NaCl solution or PBS [100].

Also, urine can be helpful in the diagnostic process of certain parasites. The specimen (5 mL) should be collected in the morning, from the “mid-stream”, into a sterile container. Genitals need to be cleaned first to avoid any debris. The patient should avoid physical exercise and maintain sexual abstinence 24 h before the collection [101]. 

**Figure 3:**
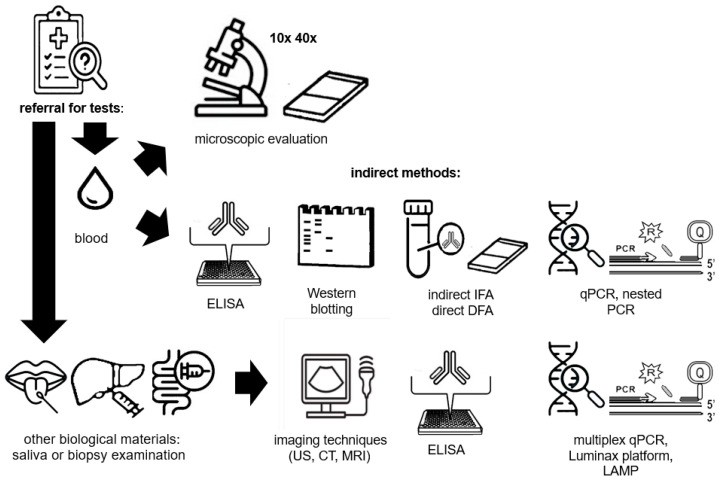
A diagram showing the procedure from referral for diagnostic tests through blood or other biological material collection to performing various diagnostic tests using direct and indirect methods. The description of the methods can be found in the text above.

A flowchart showing the procedure from referral for diagnostic tests through blood or other biological material collection to performance of various diagnostic tests using direct and indirect methods is presented in Figure 3.

### 3.3. Laboratory “Case Reports”: Examples of the Use of Molecular Methods in GIPs Diagnostics

Conventional diagnostic methods may be insufficient for diagnosing many diseases deriving from GIPs, either they because do not clearly identify the pathogen or indicate the appropriate treatment, or because they are not able to assess the possibility that new parasitic diseases may emerge in each country. Previous studies have described diagnostic procedures requiring molecular tests, as these are needed to determine the species of parasite present in a patient. This is especially important in situations where there are reports of new pathogenic threats from parasites previously considered to be non-pathogenic species.

An excellent example is an investigation the intestinal protozoan Entamoeba histolytica known to cause amebiasis, dysentery, and extra-intestinal diseases; it was recently found to co-occur with *E. dispar* and *E. moshkovskii* [102]. These findings have complicated our understanding of the pathogenic behaviors and public health importance of these nearly indistinguishable species [103,104,105,106], although many studies indicate that while *E. histolytica*, *E. dispar*, and *E. moshkovskii* are morphologically identical, they are biochemically and genetically different [107].

Various specific diagnostic tests are available for amebiasis, each with different levels of sensitivity and specificity. Wet preparation of concentrated fecal samples requires expertise in the morphology of different species of Entamoeba; the test is also nonspecific because it cannot distinguish *E. histolytica* from the non-pathogenic *E. dispar* and *E. moshkovskii* and is beset by false positives due to the misidentification of macrophages, trophozoites, polymorphonuclear leukocytes (PMN), and cysts [106]. 

Entamoeba can also be detected by culture, which is generally more sensitive than stool ova and parasite examination. However, culture techniques are time-consuming, insensitive, technically difficult, and have significant false-negative result rates; they are also often unsuccessful [108].

Isoenzyme analysis (zymodemes) of cultured amoeba also faces the same problems [109]. Several different serological assays have been developed for the detection of the specific antibodies against *Entamoeba histolytica*, including indirect hemagglutination (IHA), immunoelectrophoresis, immuno-diffusion, complement fixation, and ELISA. The only fecal antigen test that can distinguish *E. histolytica* from *E. dispar* and *E. moshkovskii* is microwell ELISA, which detects the Gal/GalNAc adherence lectin of *E. histolytica*; this test is more sensitive than stool ova and parasite examination or culture, and is rapid, taking less than two hours. However, one limitation is the need for fresh or frozen stool samples for coproantigen detection: otherwise, the detected antigens are denatured by the fixation of the stool sample. Nevertheless, this test has demonstrated good sensitivity and specificity for the detection of *E. histolytica* antigen in stool specimens of people with amoebic colitis and asymptomatic intestinal infection [110].

Since the 1990s, many clinical and epidemiological studies have described the diagnosis of amebiasis by various types of PCR; these methods are based on the differences between the small subunit rRNA gene (18S rRNA) of *Entamoeba histolytica*, *E. dispar*, and *E. moshkovskii* [110,111,112]. The tests have been approved by the WHO, especially in developed countries. The testing can be performed on various clinical specimens, such as stool, tissues, and liver abscess aspirate. Other gene targets for PCR include those coding for serine-rich *E. histolytica* protein (SREPH) and chitinase. They can also be used to amplify DNA recovered from laboratory cultures and microscopy-positive feces concentrated by the zinc–sulfate gradient flotation [109]. 

When working with amebiasis, conventional PCR can be used for the following purposes: (i) to determine the actual prevalence of the species *E. histolytica* and *E. dispar*, which is not possible using routine methods; (ii) to provide an effective diagnosis, allowing for adequate treatment of the infection [110]. 

Nested PCR provided the first report of *Entamoeba moshkovskii* infection in Bangladesh by diagnosing amoebiasis in fecal samples from children. This technique allows *Entamoeba histolytica*, *E. dispar*, and *E. moshkovskii* to be differentiated based on the size of 18S rRNA and by comparing the polymorphic sequences of the ArgTCT tRNA gene of the three species [110,111,112]. Amoebiasis can be diagnosed effectively using real-time PCR (qPCR), due to its speed, the relative quantification of the number of parasites, high sensitivity, and low risk of contamination. Multiplex qPCR protocols allow differential detection of the four *Entamoeba* species (*E. histolytica*, *E. dispar*, *E. moshkovskii*, and *E. bangladeshi*); the analysis uses primers common to all four species, with Taqman probes that hybridize with the products and differ according to the fluorescent molecules [113].

Commercial tests based on qPCR panels and TAG arrays, which use Taqman probes, can detect E. histolytica with a sensitivity of 85% and a specificity of 77% [114,115]. One kit is the Luminex platform. Testing is performed on microspheres treated with the hybridized strands that are fluorescently tagged: the beads are individually analyzed with a red laser that recognizes the microsphere set, and a green laser that indicates the bound DNA target. The Luminex beads are bound to hybridization probes that link to regions that can differentiate between *Entamoeba histolytica*, *E. moshkovskii*, *E. dispar*, *E. hartmanii*, and *E. coli*. The assay has been standardized with cloned DNA samples of each species and evaluated with 24 DNA extracts from samples obtained from individuals diagnosed with these amebiasis in their stools [113]. 

Loop-mediated isothermal amplification (LAMP) amplifies DNA with high specificity, efficiency, and rapidity under isothermal conditions. This method employs a DNA polymerase and a set of four specially designed primers that recognize a total of six distinct sequences on the target DNA. The plasticity of the method has allowed the development of qualitative variants, which can be made at a lower cost than the expensive real-time PCR technique. In addition, the polymerases used in the LAMP test are more resistant to polymerase inhibitors used in the PCR reaction, and sample preparation is simpler. In the diagnosis of amoebiasis, by amplifying regions of the 18S rRNA gene, LAMP can detect *Entamoeba histolytica* at levels of up to one parasite per reaction, with a sensitivity of 15 parasites, compared to 50 for nested PCR; it has also been found to have a specificity of 92%, making it a simple technique but with high specificity. Positive reactions for *E. histolytica* were identified as tube turbidity or staining changes using SYBR green [111,112,113,114]. 

Molecular tools have also been used to identify cystic echinococcosis (CE) caused by the metacestode stage of *Echinococcus granulosus* complex. It occurs worldwide, with the highest prevalence in parts of Eurasia (Mediterranean countries, Russia, and China), North and East Africa, Australia, and South America. Diagnosis is typically carried out using a combination of molecular and immunological approaches and comparative morphology [116,117,118]. 

The definitive diagnosis is based on the identification of protoscolices and/or hooks (hydatid sand) using light microscopy in biopsy or surgically removed material. Microscopy is simple, rapid, and quite cheap, but it is not very useful when working with a low number of parasites or in cases where the species are morphologically identical. In some cases, microscopic evaluation is impossible due to the cyst membrane degenerating in response to inter alia host defense mechanisms, spontaneous rupture, or aging. In such cases, indirect diagnostic tools such as immunological and molecular methods can be valuable [119,120]. One common diagnostic method for the *Echinococcus* spp. is serological testing: the ELISA test is used as a basic examination, followed by confirmation with the immunoblot assay. However, due to its limited sensitivity and specificity, immunodiagnostics should be regarded as a diagnostic aid. A significant number of infected patients do not produce an immune response, which translates into a significant percentage of false-negative results [121,122]. 

In echinococcosis, the use of molecular diagnostics may represent a good example of avoiding many problems associated with conventional diagnostics. This technique may constitute the gold standard in the diagnosis of these parasites [122]. Genetic studies of *Echinococcus* have been conducted based on sequence data from mitochondrial genes encoding cytochrome c oxidase subunit I (cox1), NADH dehydrogenase I (nad1), and ATPase 6 (atp6), and the nuclear rDNA internal transcribed spacer 1 (its1) [123]. Studies of the mitochondrial and nuclear genes of different *Echinococcus* species has led to their taxonomic revision: the genotypes G1–G3 are now grouped as *E. granulossus sensu stricto*, G4 as *Echinococcus equinus*, G5 as *Echinococcus ortleppi*, and G6–G10 as *Echinococcus canadensis*, and the “lion strain” is an *E. felidis* [124,125,126]. 

Dybicz et al. (2013) examined fresh tissue samples collected from 47 patients after hepatectomy, between 2000 and 2010 in the Department of General, Transplant, and Liver Surgery, Medical University of Warsaw, Poland. All patients were suspected of having CE, although in some cases, this was not confirmed by immunological testing (ELISA) or imaging techniques (US, CT). The observation of protoscoleces and hooks in wet sample preparations under light microscopy confirmed 27 samples as positive for CE. The PCR analysis confirmed the presence of *Echinococcus* species in 30 cases, including 3 found to be negative by microscopy; and nad1 sequence alignments showed identity with the G7 (pig) strain, *Echinococcus canadensis*. Thus, these results indicate that 30 of the 47 examined patients (64%) had CE; this had a significant influence on the management of their treatment [127]. After the amplification of the same mitochondrial DNA fragments, Dybicz et al. identified *E. granulosus* G1 in seven human liver cysts; this included the first report of human cystic echinococcosis caused by *E. ortleppi* in Poland. This species was previously regarded as extinct, as the most frequent causative agent of human CE in Poland is *E. canadensis* G7 [127,128,129].

Many other diagnostic tests use genotyping methods which require a lot of time or expense. To simplify the genotyping of the *Echinococcus granulosus* isolates, Boubaker et al. designed a single-tube multiplex PCR (mPCR). Briefly, part of the *E. granulosus* complex (G1, G5, G6/G7) can be genotyped by consecutive PCR, making further discrimination possible between *E. multilocularis*, *E. granulosus* s.s. (G1), and an *E. ortleppi* (G5)/E. canadensis (G6/G7) cluster [129,130]. 

Parallel PCR approaches can be combined in a multiplex PCR setup. Thees approaches have been successfully applied worldwide in many aspects of DNA analyses, especially in the molecular diagnosis of infectious diseases [131]. 

In 2019, Cinzia Santucciu et al. designed and validated the “one-step-PCR” diagnostic protocol for the identification of *E. granulosus* and the identification of its genotype (G1–G3). The PCR technique, named PCR *E.g.*s.s., detects *E. granulosus* s.s., through the amplification of the 1001 bp *Cal* gene, coding for Calreticulin l. The amplicon is distinctive of genus, species, and genotype of *E. granulosus* G1–G3. This diagnostic technique avoids the need to sequence the DNA sample; hence, it is a simple and quick method for diagnosis in humans and animals, allowing “one-step detection” of the pathogenic agent in routine practice [132].

### 3.4. Possibilities of Using Artificial Intelligence to Increase the Effectiveness of Diagnosing Parasitic Diseases

The healthcare sector has always prioritized the response to and management of infectious diseases. Human exposure to and experience with epidemics and pandemics, including the recent COVID-19 pandemic, have shown the dire need to develop a more resilient healthcare ecosystem [133,134,135]. Currently, global warming facilitates the geographical spread of parasites, especially those transmitted by vectors [136,137,138].

Artificial intelligence (AI), through the power of machine learning (ML) and deep learning (DL) algorithms, is likely to revolutionize parasite diagnostics in the near future, offering faster, more accurate, and more accessible solutions. Its algorithms can be trained on large datasets of parasite images, allowing them to identify specific features and morphological patterns with extreme accuracy in the future. AI predictive algorithms can help understand parasite transmission and epidemic patterns by analyzing epidemiological data, environmental trends, and population statistics. This will allow for improved public health interventions, resource allocation, and outbreak-prevention strategies, which may prevent the spread of disease. AI, although yet not routinely used in laboratories, is already finding initial applications for accurate and rapid identification of parasites through mechanical image analysis [139]. The utility of AI in detecting intestinal parasitic products such as cysts or eggs in stool samples has been investigated. It has been found that the use of AI models can improve the diagnosis of parasites in trichrome-stained stool samples (such as *Giardia lamblia*, *Entamoeba* spp., or *Blastocystis* spp.) in clinical settings [140,141]. After preparing digitized microscopic slides of stool samples, the pre-trained AI model showed more than 98% agreement with manual examinations performed by expert parasitologists. This model can not only perform the examination autonomously but also highlight suspicious areas in the images for further manual analysis and confirmation, thus improving the standard diagnostic process. A similar approach has been shown to be effective in detecting several intestinal helminth cysts and eggs, including roundworms, *Taenia* spp., and *A. lumbricoides* [142]. It is worth mentioning that even microscopic images captured by a smartphone camera, augmented with open-access tools, yielded impressive results. Even with a training dataset consisting of less than 4000 images from five typed specimens, the developed ML models based on the You Only Look Once (YOLO) detection model achieved an accuracy of around 97%, with detection times of only 8.5 and 15.4 milliseconds. In another study, the YOLO model trained on a smaller sample of 1773 images from 34 common classes of intestinal helminth products still showed precision and sensitivity exceeding 95% [143]. AI has also been applied to capsule endoscopy (CE) images, which has improved the detection of small intestinal parasitic diseases such as ancylostomiasis. A deep CNN system based on the YOLO-V4 (You Look Only Once-Version4) detector using 11,236 CE images of nematodes achieved more than 91% sensitivity, specificity, and accuracy in detecting nematodes in CE images and even identified additional real nematodes that were previously missed by experts. The authors are already developing an improved version of this model, incorporating light amplification technology to mitigate the effects of darkness [144]. However, to identify *E. multilocularis* when its larvae infiltrate the liver parenchyma, a different approach is needed: in such cases, diagnosis is based on histological sections of liver tissue. ML and deep learning (DL) models have also shown great potential here, resulting in 98% accuracy and precision. Similar results were reported for another DL model used for the diagnosis of Echinococcus spp. in liver ultrasound imaging [145]. As mentioned earlier, images for AI models do not necessarily need to be captured using professional or expensive microscope cameras. With easy access to various enhancement tools, even smartphone cameras can be used effectively. Furthermore, many auxiliary components, such as optical accessories for lens-less, brightfield, or fluorescence imaging, can be 3D-printed [146]. The steady progress in both AI and tools that facilitate point-of-care medicine gives great promise for improving diagnostics, including the routine diagnosis of parasitic diseases. This is the future, but it is—most likely—not far away.

## 4. Conclusions

Many parasites that once posed a challenge to the poorest countries have adapted to climate change and are found in many countries around the world. Therefore, innovations and health practices are needed to effectively respond to these global biological threats. A crucial role in identifying parasites, and thus indicating appropriate treatment, is played by laboratory diagnostic tests. Correct laboratory diagnosis of GIP is not easy. It is based on the use of various biological materials and diverse methodologies, including traditional microscopic methods and modern molecular methodologies. GIP diagnostics involves not only determining the presence of the parasite but also establishing the relationship between the parasite invasion and disease symptoms. The diagnostic process should consider the possibility of coexistence of infection with several parasites at the same time. In such a situation, diagnostics should be planned with consideration of their frequencies in each population and local epidemiological situations. The importance of the proper interpretation of laboratory test results, based on a good knowledge of the biology of parasites, should be emphasized. Artificial intelligence is playing an increasingly important role in the healthcare ecosystem, greatly helping to improve diagnosis, treatment, and monitoring of patients. Artificial intelligence is very useful in processing and interpreting large amounts of images of blood smears, stool samples, and tissue biopsies. Using machine learning tools such as CNN, AI is useful for identifying forms of parasites such as eggs, larvae, and adult worms. Many studies have proven the diagnostic power of artificial intelligence in detecting various intestinal parasites in stool samples, such as hookworms, roundworms, and whipworms, as well as schistosomiasis. Artificial intelligence not only offers the benefits of high sensitivity, but also has a much lower detection limit than conventional strategies while significantly reducing the incidence of human error and workload.

## Data Availability

The data presented in this study are available in the cited articles that are listed in the reference list.
